# HIV envelope trimer-elicited autologous neutralizing antibodies bind a region overlapping the N332 glycan supersite

**DOI:** 10.1126/sciadv.aba0512

**Published:** 2020-06-05

**Authors:** Bartek Nogal, Laura E. McCoy, Marit J. van Gils, Christopher A. Cottrell, James E. Voss, Raiees Andrabi, Matthias Pauthner, Chi-Hui Liang, Terrence Messmer, Rebecca Nedellec, Mia Shin, Hannah L. Turner, Gabriel Ozorowski, Rogier W. Sanders, Dennis R. Burton, Andrew B. Ward

**Affiliations:** 1 Department of Integrative Structural and Computational Biology, The Scripps Research Institute, La Jolla, CA 92037, USA.; 2 Division of Infection and Immunity, University College London, London WC1E 6BT, UK.; 3 Department of Medical Microbiology, Amsterdam UMC, Location AMC, University of Amsterdam, Amsterdam 1105AZ, Netherlands.; 4 Department of Immunology and Microbiology, The Scripps Research Institute, La Jolla, CA 92037, USA.; 5 International AIDS Vaccine Initiative–Neutralizing Antibody Center (IAVI-NAC), The Scripps Research Institute, La Jolla, CA 92037, USA.; 6 Center for HIV/AIDS Vaccine Development (CHAVD), The Scripps Research Institute, La Jolla, CA 92037, USA.; 7 Ragon Institute of MGH, MIT and Harvard, Cambridge, MA 02139, USA.

## Abstract

To date, immunization studies of rabbits with the BG505 SOSIP.664 HIV envelope glycoprotein trimers have revealed the 241/289 glycan hole as the dominant neutralizing antibody epitope. Here, we isolated monoclonal antibodies from a rabbit that did not exhibit glycan hole–dependent autologous serum neutralization. The antibodies did not compete with a previously isolated glycan hole–specific antibody but did compete with N332 glycan supersite broadly neutralizing antibodies. A 3.5-Å cryoEM structure of one of the antibodies in complex with the BG505 SOSIP.v5.2 trimer demonstrated that while the epitope recognized overlapped the N332 glycan supersite by contacting the GDIR motif at the base of V3, primary contacts were located in the variable V1 loop. These data suggest that strain-specific responses to V1 may interfere with broadly neutralizing responses to the N332 glycan supersite and vaccine immunogens may require engineering to minimize these off-target responses or steer them toward a more desirable pathway.

## Teaser

Strain-specific autologous neutralizing antibodies may compete with N332 supersite targeting broadly neutralizing precursors.

## INTRODUCTION

Given their protective efficacy in passive transfer studies, the elicitation of broadly neutralizing antibodies (bnAbs) is one of the primary objectives of current HIV research (*1*–*6*). Stabilized envelope glycoprotein (Env) SOSIP trimers contain all bnAb epitopes aside from the membrane-proximal external region and have provided a platform for elicitation of such bnAb responses (*7*–*10*). These stabilized SOSIP immunogens yield neutralization titers against immunogen-matched difficult-to-neutralize (Tier-2) viruses in many animals (*10*–*17*). Previous studies (*14*, *18*) and more recent imaging of polyclonal antibody responses (*19*) revealed that the primary target of neutralization on BG505 induced in rabbits is an epitope within a hole in the glycan shield. This glycan hole (GH) is mostly specific to the BG505 strain and includes a missing glycan at position N241, which is conserved in >97% of circulating Env strains (www.hiv.lanl.gov), and therefore, there is very limited potential for broadening such responses. Bioinformatic analyses have indicated that GHs are commonly found in many HIV strains, although at different positions. As illustrated by the BG505 strain, they can involve the absence of relatively highly conserved potential N-linked glycosylation sites (*18*, *20*). Overall, these studies suggest that GHs are immunogenic sites that induce strain-specific nAbs after both infection and immunization, that neutralization escape from these antibodies is relatively easy, and that the corresponding responses are not on the pathway to bnAbs.

Given the limited potential of GH antibodies to develop broader reactivity, we sought to identify and characterize GH-independent nAbs that arose in BG505 SOSIP.664–immunized rabbits to bring about a more extensive understanding of how autologous neutralization arises and to evaluate the potential for broader responses among these alternative epitopes (*14*). Here, we describe the high-resolution cryo–electron microscopy (cryoEM) structure of a BG505 nAb that binds to an epitope that is composed of the variable V1 loop and that overlaps substantially with the well-known N332 glycan supersite epitope on the high-mannose patch of the outer domain of gp120 (*21*–*23*). We conclude that these new antibodies likely do not have the potential to recognize a broad set of Envs and are potentially further problematic in their competition with bona fide bnAbs such as PGT128, PGT121, PGT135, and BG18 (*24*–*27*), making it unlikely that N332-targeting antibodies can be elicited when these V1-targeting antibodies appear.

## RESULTS

### Trimer-elicited mAbs potently neutralize BG505 and very closely related viruses

**Fig. 1:**
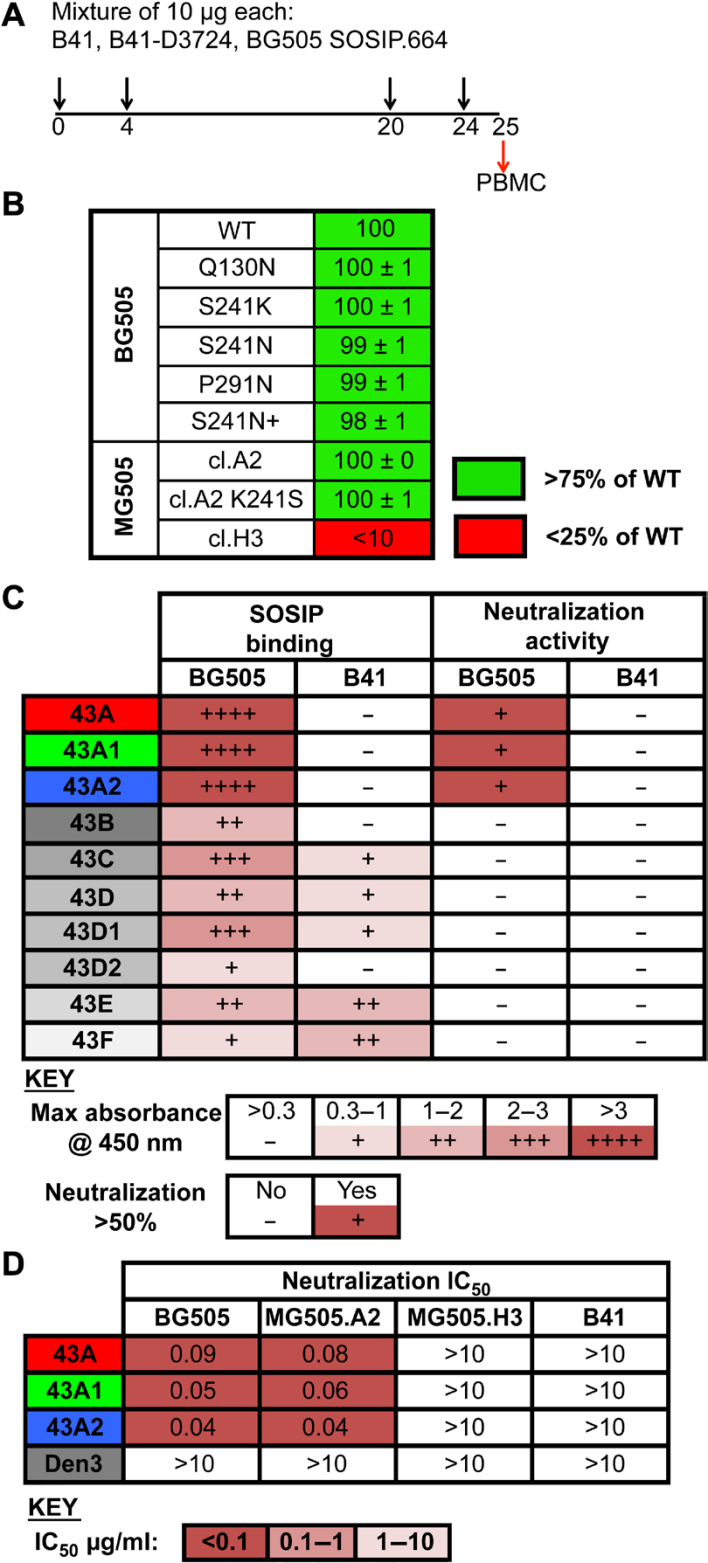
43A family antibodies neutralize BG505 and MG505 viruses. (**A**) Immunization scheme for rabbit 5743 as previously described (*14*). PBMCs were harvested 1 week following the final immunization, and immunogen-specific B cells were isolated by fluorescence-activated cell sorting (FACS) as detailed in Materials and Methods. (**B**) The first two columns show a qualitative comparison (see key) of binding by 10 crude unpurified mAbs to the indicated Avi-tagged biotinylated immunogens, which were captured on streptavidin-coated plates. Rabbit mAb binding was detected with alkaline phosphatase–conjugated mouse anti-rabbit IgG. The second two columns show whether these unpurified mAb preparations could neutralize the indicated pseudoviruses in the TZM-bl assay. (**C**) Purified neutralizing mAbs were titrated against the indicated pseudoviruses to generate the IC_50_ values shown. (**D**) Week 22 rabbit 5743 serum neutralization of pseudoviruses with indicated point mutations in the TZM-Bl assay (*14*). WT, wild type.

A previous study reported the immunogenicity of BG505 and B41 SOSIP.664 immunogens in rabbits, including coimmunization with both immunogens, and serology indicated neutralizing responses outside of the GH epitope (Fig. 1A) (*14*). From this cohort, the post-immune plasma from 14 rabbits (*14*) were titrated against pseudoviruses derived from the MG505-BG505 mother-to-child transmission pair (*28*). Four rabbits were found to lack neutralizing activity against the previously described immunodominant GH in BG505, because no gain of function was observed against the MG505.A2 K241S pseudovirus relative to wild-type MG505.A2 in the TZM-bl neutralization assay (*14*). S241 is a critical contact residue in the center of the GH epitope that, when mutated to a lysine, which naturally occurred in MG505.A2, eliminates GH-based neutralization. One animal, rabbit 5743, not only lacked GH-dependent neutralization but also had high-titer neutralization activity against the wild-type MG505.A2 virus (Fig. 1B). Therefore, cryopreserved primary blood mononuclear cells (PBMCs) isolated from rabbit 5743 1 week after the final immunization were used to sort BG505-specific B cells. Single B cell cloning (*18*) resulted in the isolation of 12 monoclonal antibodies (mAbs) from rabbit 5743, three of which neutralized the BG505, MG505.A2, and MG505.A2 K241S pseudoviruses, recapitulating the plasma neutralization activity of the source rabbit (Fig. 1, B to D). These three mAbs were named 43A, 43A1, and 43A2 as they are somatic variants sharing a common VDJ recombination event, although 43A2 has likely undergone a subsequent gene conversion event and thus uses a distinct VH gene (fig. S1).

### 43A family antibodies bind a non-GH epitope

**Fig. 2:**
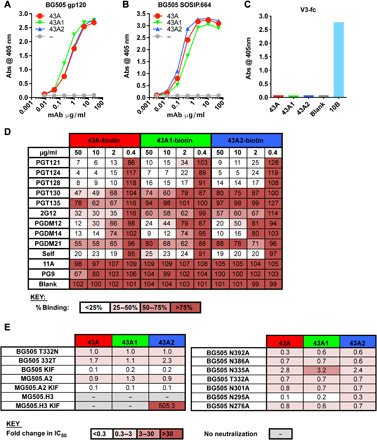
43A family antibodies bind to an epitope overlapping the bnAb N332 glycan supersite. Dose-response binding of neutralizing rabbit mAbs indicated in the legend was assayed by ELISA using streptavidin-coated plates to capture Avi-tagged biotinylated BG505 (**A**) gp120 or (**B**) SOSIP.664. (**C**) Binding by the indicated rabbit mAbs was assayed by ELISA using V3-Fc–coated plates, where the previously described V3 rabbit mAb 10B (*18*) was used as a positive control. (**D**) Competitor nonbiotinylated mAbs listed in the right-hand column were preincubated with Avi-tagged BG505 SOSIP.664 protein. Binding of the biotinylated mAbs listed across the top row is expressed as percent binding, where 100% was the absorbance measured in the absence of a competitor (blank). % Binding is color-coded according to the key. (**E**) The rabbit mAbs were titrated against the pseudovirus mutants indicated in the TZM-bl assay, and IC_50_ values were calculated using GraphPad Prism and fold change relative to the immunogen-matched strain (BG505 T332N).

All 43A mAbs were found to bind to the gp120 subunit of the BG505 by enzyme-linked immunosorbent assay (ELISA; Fig. 2, A and B) but did not react with the peptide of the V3 loop of BG505 on an Fc scaffold (Fig. 2C). Notably, none of the mAbs bound to the other immunogen were administered during the immunization study, i.e., B41 SOSIP.664 (*14*) (Fig. 1A). All 43A mAbs were found to compete with one another for binding to BG505 SOSIP.664 trimer by ELISA (Fig. 2D). We also did not observe competition with the previously isolated BG505 GH-specific mAb 11A (*18*) (Fig. 2D). Moreover, the mAbs strongly competed with binding by the V3 glycan supersite bnAbs PGT121, PGT124, PGT128, 2G12, PGDM12, and PGDM14 and, to a lesser degree, by PGT130 and PGDM21 (Fig. 2D). In light of competition with these glycan-specific bnAbs, we tested the ability of the 43A antibodies to bind to a glycan array; however, no binding was detected even at high concentrations of mAb. In addition, no change in neutralization activity was observed when any of the N332 region glycan sites at positions 295, 301, 332, 386, or 392 were eliminated from the BG505 pseudovirus (Fig. 2E). The potency of neutralization did, however, increase when the virus was grown in the presence of kifunensine, which results in relatively homogeneous Man9 glycans on the Env protein. Overall, these data suggest that the 43A mAbs primarily interact with the protein amino acids and not the glycans themselves.

### 43A mAbs neutralize MG505.A2 but not MG505.H3

**Fig. 3:**
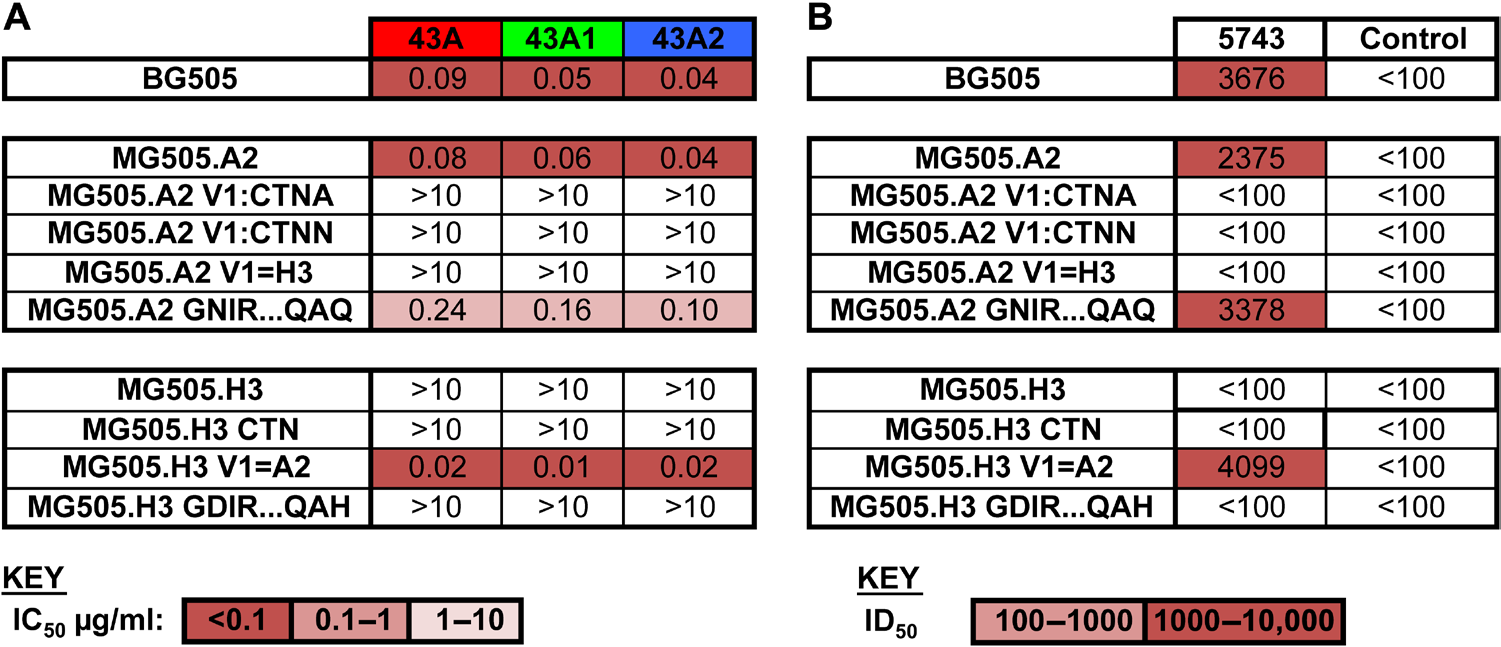
V1 loop in MG505.H3 is not compatible with 43A binding. (**A**) The rabbit mAbs were titrated against the pseudovirus mutants indicated in the TZM-bl assay, and IC_50_ values were calculated using GraphPad Prism. (**B**) Plasma from 5743 at week 25 and from an unimmunized rabbit (control) were titrated against the pseudovirus mutants indicated in the TZM-bl assay. ID_50_ (50% reciprocal dilution titers) were calculated in GraphPad Prism. ID_50_ and IC_50_ values are color-coded according to the keys below each table.

Neutralization activity of the 43A mAbs was restricted to the immunogen-matched strain BG505 and the closely related MG505.A2 virus but did not neutralize an additional virus, MG505.H3, derived from the mother. There are 13 differences in Env between MG505.A2 and MG505.H3 at positions highlighted in fig. S2. To identify which changes abrogated neutralization of MG505.H3, the 43A mAbs were tested against a panel of MG505.A2 mutant viruses where variant positions were altered to encode the MG505.H3 sequence in turn. There are two amino acid changes in the base of the V3 loop between MG505.A2 (G**D**IRQA**Q**) and MG505.H3 (G**N**IRQA**H**), but altering these, either alone or in combination, had a minor (~2.5-fold) effect on the neutralization activity of the 43A mAbs (Fig. 3A). The only change that prevented neutralization by the 43A mAbs was the introduction of an extra asparagine within the V1 loop of MG505.A2 at residue 133 (CTNN) to mimic that found in MG505.H3 (Fig. 3A and fig. S2C). The introduction of an alanine at the same position (CTNA) also blocked neutralization by all three mAbs (Fig. 3A), indicating that the loop insertion, rather than amino acid identity, is the primary factor in escape from neutralization activity. To confirm this specificity for the V1 loop sequence in MG505.H3, two additional virus mutants were produced where the V1 loops were swapped so that MG505.A2 encoded the full V1 of MG505.H3 and vice versa. The presence of the V1 from MG505.H3 prevented all neutralization, while the presence of the V1 from MG505.A2 allowed neutralization of MG505.H3 (Fig. 3A).

Notably, neutralizing responses targeting the epitope bound by the 43A mAbs dominated in the source rabbit serum sample (Fig. 3B) and similar serum specificities have recently been observed following nonhuman primate (NHP) immunization with BG505 Env (*16*). Three of the nine NHPs with the best BG505 neutralizing responses produced post-immune sera that could neutralize MG505.A2, but not MG505.H3 (*16*). These serum samples were then tested against the V1 loop swap viruses described here, and all activity was lost when the MG505.H3 V1 loop was inserted into MG505.A2 (*16*) as seen for the 43A mAbs, although the precise epitope targeted was not revealed in this study.

### EM studies reveal the details of 43A mAb epitopes

**Fig. 4:**
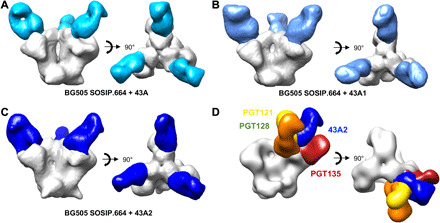
43A family antibodies all bind the gp120 at a similar angle to human bnAbs. Negative-stain EM 3D reconstructions of (**A**) 43A, (**B**) 43A1, and (**C**) 43A2 each in complex with BG505 SOSIP.664. The Fabs are colored in shades of blue, and the Env trimer is colored in white. (**D**) Comparison of 43A2 with N332 glycan supersite bnAbs. For clarity, Fabs bound to one protomer only are shown. Side views are on the left-hand side and top views on the right-hand side of each panel.

To confirm the epitopes identified by viral mutagenesis and binding competition assays, all three 43A mAbs were digested into Fab and complexed with the BG505 SOSIP.664 immunogen and then visualized by negative-stain single-particle EM (Fig. 4). The resulting three-dimensional (3D) reconstructions revealed that all three neutralizing mAbs have highly overlapping epitopes near the base of V3 and similar angles of approach as previously characterized bnAbs (Fig. 4D).

**Fig. 5:**
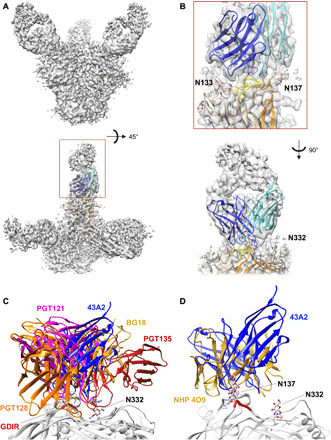
CryoEM reconstruction of 43A2 bound to BG505 SOSIP.v5.2. (**A**) CryoEM map of the BG505 SOSIP.v5.2 trimer bound three 43A2 Fabs. (**B**) Chimera-Modeller–generated 43A2 model showing how the antibody nestles itself within the surrounding glycans of the V1 loop (upper) as well as the N332 glycan supersite of vulnerability (lower). (**C**) Overlay with glycan supersite bnAbs PGT128 (orange, PDB: 5C7K), PGT121 (magenta, PDB: 5CEZ), BG18 (gold, PDB: 6CH7), and PGT135 (firebrick, PDB: 4JM2) showing less clashing between 43A2 and PGT135 with PGT121, PGT128, and BG18. (**D**) Overlay of 43A2 and NHP V1 loop-binding polyclonal antibodies from animal 4O9, showing similar focus on V1 loop interaction over N332 (*32*).

To elucidate the molecular details of the epitopic region, the most potent antibody, mAb 43A2, was complexed with the BG505 SOSIP.v5.2 trimer and subjected to single-particle cryoEM analysis, where we obtained a high-resolution reconstruction at ~3.5-Å global resolution (Fig. 5A, fig. S3, and table S1). This map enabled building and refinement of an atomic resolution model of the complex. The model revealed that, although the 43A2 mAb contained a 13–amino acid–long CDRL3 that is inserted into the gp120 N332 glycan supersite region, the primary molecular contacts are with V1 of gp120. Leu^94^ and Asp^95^ of the CDRL3 make peptide backbone contacts with gp120 Ile^138^ and Asn^136^, respectively, while avoiding interaction with glycans at positions 137 and 133 (Fig. 5B and fig. S4, A and B). There is very little interaction with any of the surrounding glycans, consistent with the neutralization data for glycan knockout viruses (Fig. 2, D and E). Further interaction with the V1 loop is achieved via 43A2 CDRH3 Gly^99^ and Ser^100^ with the side chains of gp120 Asp^140^ and Asp^141^ (fig. S4, C and D). The insertion of an amino acid in V1 resulting in loss of neutralization, such as observed in residue 133 of MG505.H3 (Fig. 3A), would change the registration of the downstream V1 residues and therefore potentially disrupt interactions with Asp^140^ and Asp^141^ or result in a steric clash with the antibody.

In addition to the extensive contact with V1, Leu^94^ of CDRL3 does contact Asp^325^ of the conserved gp120 GDIR coreceptor-binding motif (fig. S4E), consistent with the reduced neutralization of the GNIR mutant (Fig. 3A). However, when compared to the epitopes of PGT135, PGT121, PGT128, and BG18, the bnAbs make more extensive contacts through their extended ~20–amino acid CDRH3s compared to the 13–amino acid CDRL3 of 43A2 (Fig. 5C). Notably, there is substantial overlap between 43A2 and PGT128, where both the 43A2 CDRL3 and PGT128 CDRH3 compete for the same residue 325 that is part of the GDIR motif (fig. S4E). In contrast, the weaker competition observed between PGT135 and 43A2 in the ELISA may be explained by the lack of engagement by the PGT135 bnAb with residues in the GDIR motif (Fig. 5C).

**Fig. 6:**
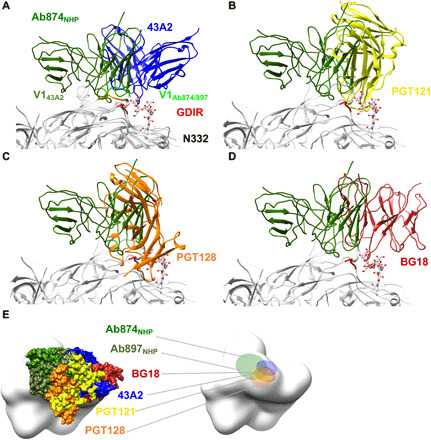
Comparison of V3 glycan-targeting bnAbs and 43A2 with nonneutralizing NHP antibodies 874 and 897 (*35*). (**A**) The V1 loop adopts an “up” conformation, when Ab_874NHP_ and Ab_897NHP_ are bound. (**B** to **D**) Binding pose of Ab_874NHP_ with respect to bnAbs. (**E**) Ab_874NHP_ and Ab_897NHP_ footprint relative to bnAbs and 43A2, showing that the two non-nAbs bind considerably more distant from N332 glycan supersite bnAbs.

The 43A2-bound BG505 V1 loop remains in the ground-state conformation, which we define as the conformation observed in structures of Env that do not have an antibody bound to the N332 supersite or V2 apex epitopes, which influence the V1 conformation (*29*–*31*). The ground-state conformation was also observed in the NHP V1-specific antibody revealed in a polyclonal imaging approach described elsewhere (Fig. 5D and fig. S4F) (*21*, *30*, *32*). In bnAb structures, the V1 loop is lifted up, providing greater access to the GDIR motif (*25*, *33*). We postulate that the ground-state conformation of V1 prevents additional interaction with the GDIR motif and therefore is an impediment to accessing the full extent of this important bnAb site. Further, even if the V1 loop is predisposed to an “up” conformation as inherent to N332 supersite germline-targeting immunogens, there are still many alternative binding poses of antibodies that likely represent off-target responses (Fig. 6) (*34*, *35*).

## DISCUSSION

Previous studies revealed that approximately 25% of rabbits immunized with BG505 SOSIP produce neutralizing responses outside of the GH epitope (*18*, *36*), while, in NHPs, the number was higher (*16*). To probe details of these responses, we isolated mAbs from one such rabbit, 5743 (*14*), and subjected the antibodies to antigenic and structural characterization. It is unclear why rabbit 5743 responded differently to an immunization protocol, which in three other animals produced GH-dependent responses, because all animals were cohoused and immunized at 4 months of age, limiting the potential for environmental differences. However, New Zealand White (NZW) rabbits are an outbred strain and, as such, it is plausible that genetic differences predisposed rabbit 5743 to react to this epitope.

Our original aim was to generate a deeper understanding of how strain-specific neutralization occurs and establish if potential for broader responses exists among these alternative epitopes. This is an important goal given the apparently limited scope to broaden the previously described GH-specific response (*18*, *19*, *36*). In addition, recent studies have revealed that the GH around positions 241/289 appears to be less immunodominant in nonhuman NHP immunization (*16*). Thus, our study describes a mAb that targets a neutralizing epitope in V1 following immunization of two different species. While the epitope footprint resembles the N332 bnAb supersite, there are several key differences, and although it may be possible to broaden these V1 responses by increasing interaction with the more conserved GDIR motif, there are several hurdles that need to be overcome. Thus, we conclude that the V1 is a hotspot for strain-specific antibodies that are likely to compete with the N332 bnAb supersite epitope.

Current understanding of how human bnAbs bind the glycan supersite suggests that there are two ways nAbs attack this area to achieve breadth: (i) bind directly to conserved glycans, such as N332, and (ii) bind to the conserved GDIR motif by inducing a conformational change in the V1 loop that typically restricts access to the site. PGT128, PGT121, and BG18 are examples of bnAbs that use both mechanisms (*21*, *22*, *27*). In contrast to bnAbs, the neutralization activity of the rabbit 43A mAbs is not adversely affected by the removal of any particular glycan, nor is there evidence of specific glycan contacts in our cryoEM map. Furthermore, virus produced with artificially homogenous Man9 glycans is more potently neutralized, which, along with our cryoEM data, suggests that the underlying peptide is better exposed when glycans are more homogeneous. Thus, while the 43A mAbs have effectively navigated the glycan shield near the high-mannose patch on the gp120 outer domain, they are biased toward the variable V1 loop and are not capable of providing broad neutralization.

Given our results, we conclude that elicitation of bnAbs against the N332 glycan supersite using wild-type Env sequences remains a substantial challenge. Strain-specific nAbs do not typically include bnAb features like long CDRH3 loops, primarily bind to epitopes composed of peptide only, present lower bars to elicitation than bnAbs, and can be highly competitive with bnAbs. Thus, immunogens that are designed to exquisitely stimulate appropriate precursors and then shepherd responses along narrow pathways to bnAbs may be required. Such an approach has been shown effective in heavy chain knock-in mice with heavily biased precursor repertoires (*34*, *37*). However, in more realistic situations, such as human vaccination, involving greater competition between antibody responses, we hypothesize that the achievement of broad neutralization will be substantially more challenging. Of note, a recent study reported the elicitation of antibodies to the N332 supersite epitope in wild-type mice and NHPs (*35*). However, despite biasing the immune response towards the N332 epitope by removing a conserved glycan (N156) from the original germline-targeting immunogen (*34*, *37*), the elicited antibodies were not neutralizing. Structures of three antibodies (*35*) in complex with the cognate immunogens revealed some weak similarity to the recognition mode of bnAbs, but the antibodies lacked the hallmarks of bnAbs including a long HCDR3 and explicit recognition of conserved glycans (Fig. 6, fig. S5, and table S2), similar to nAb 43A. The antibodies did bind with the V1 in the up conformation (Fig. 6A and fig. S5D), which is likely predisposed to this conformation by the N136P mutation (fig. S5D) introduced in the original GT trimer (*34*, *37*). However, because of the removal of glycans used in the immunogen design, the antibodies were located relatively far from the core of the bnAb epitope, even farther than the 43A nAbs (Fig. 6, B to E). The NHP antibodies also made specific contacts with several non-native, engineered mutations as well as the N156Q mutation introduced to remove the glycan (table S2). It is therefore difficult to see how the NHP antibodies and their corresponding binding pose, and contact with N156Q, could be further matured to recognize an intact glycan shield and cross-react with a large diversity of Envs. Thus, glycans play a key role in steering immune responses toward the correct footprints and binding poses.

Overall, although it appears possible to target features of the N332 supersite in different models (table S2) using available immunogens, substantial hurdles remain to eliciting authentic bnAbs to this site. Although these strain-specific nAbs target an overlapping epitope, the details of the molecular interactions are quite different, and more worryingly, they can compete with antibodies that may have higher probability to evolve into broad neutralization, and thereby potentially suppress bnAb responses.

## MATERIALS AND METHODS

### Experimental design

Immunizations of female NZW rabbits were carried out as previously described (*14*). The study was carried out in accordance with protocols approved by the Institutional Animal Care and Use Committee at Covance Research Products Inc. (Denver, PA), with approval number C0014-15. The protocols were in compliance with the Animal Welfare Act and other federal statutes and regulations relating to animals and adhered to the *Guide for the Care and Use of Laboratory Animals* (National Research Council, 1996). Rabbit 5743 serum exhibited strong autologous neutralization of the BG505 pseudovirus, but unlike neutralizing sera of most rabbits, 5743 also neutralized the closely related MG505.A2 strain, where a lysine at position 241 abrogates GH neutralization. Thus, the objective of this study was to characterize and map the response(s) exhibited by rabbit 5743 serum. Toward that end, we isolated PBMCs and performed BG505-specific B cell sorting to isolate the relevant mAbs. Subsequently, site-directed mutagenesis of pseudoviruses and ELISAs were used to approximate the BG505 epitope to which the 5743 mAbs were elicited, and whether these isolated mAbs overlap with known bnAbs. To determine the degree to which glycans were involved in the paratope-epitope interactions, we also performed neutralization assays with deglycosylated BG505 and MG505.A2 pseudoviruses. To visually confirm the epitope of the 43A class of mAbs, negative-stain EM was used, followed by high-resolution cryoEM with 43A2 to elucidate the details of the BG505 epitope–43A2 paratope toward informing immunogen design.

### Isolation of rabbit B cells

Cryopreserved PBMCs from rabbit 5743 were thawed, resuspended in 10 ml of RPMI 10% fetal calf serum (FCS), and collected by centrifugation at 600*g* for 5 min. Cells were washed with phosphate-buffered saline (PBS), resuspended in 10 ml of PBS, and collected by a second centrifugation step. Cells were resuspended in 100 μl of FWB (2% FCS–PBS) with anti-rabbit immunoglobulin M (IgM) fluorescein isothiocyanate (FITC) (1:1000) and a streptavidin-allophycocyanin (APC) tetramer of biotinylated anti-rabbit IgG. After 1 hour on ice, cells were washed once with 10 ml of PBS, collected by centrifugation, and resuspended in 100 μl of FWB with 1 μl of a streptavidin-phycoerythrin (PE) tetramer of biotinylated BG505 or B41 SOSIP.664. After a further 1 hour on ice, cells were washed once with 10 ml of PBS, collected by centrifugation, and resuspended in 500 μl of FWB for sorting on BD FACSAria III. IgM^−^IgG^+^BG505^+^B41 lymphocytes were collected at one cell per well into SuperScript III Reverse Transcriptase lysis buffer (Invitrogen) as previously described and immediately stored at −80°C before complementary DNA generation and single-cell polymerase chain reaction.

### Generation of antibodies and Fabs

Rabbit antibody variable regions (GenBank accession number: KX571250-1324) were cloned into an expression plasmid adapted from the pFUSE-rIgG-Fc and pFUSE2-CLIg-rK2 vectors (InvivoGen). Human and rabbit antibodies were transiently expressed with the FreeStyle 293 Expression System (Invitrogen). Antibodies were purified using affinity chromatography (Protein A Sepharose Fast Flow, GE Healthcare), and the purity and integrity were checked by SDS–polyacrylamide gel electrophoresis. To generate Fabs, rabbit IgG was digested with 2% papain (Sigma, P3125) in digestion buffer [10 mM l-cysteine, 100 mM Na acetate (pH 5.6), 0.3 mM EDTA] for 6 hours and then quenched with 30 mM iodoacetamide. Undigested IgG and Fc fragments were removed by affinity chromatography, and the Fab-containing flow through was collected. Size-exclusion chromatography was performed using Superdex 200 10/300 resin (GE Healthcare) to remove papain and digestion by-products.

### Neutralization assays

Pseudovirus neutralization assays using TZM-bl target cells were carried out as previously described (*38*). Briefly, single-round infectious HIV Env pseudoviruses were made as described previously (*39*). Briefly, plasmids encoding Env were cotransfected with an Env-deficient backbone plasmid (pSG3DENV) using FuGENE 6 (Promega). Virus-containing supernatants were harvested 48 hours after transfection, stored at −80**°**C, and then titrated on TZM-bl target cells to determine the dilution needed for neutralization assays. Filter-sterilized mAbs and/or plasma were then serially diluted in a 96-well plate and incubated with virus for 1 hour before the addition of TZM-bl target cells. After 48 hours, the relative light units (RLU) for each well were measured, and neutralization was calculated as the decrease in RLU relative to virus-only control wells. ID_50_/IC_50_ values reported as the reciprocal dilution/antibody concentration that resulted in 50% virus neutralization after fitting the curve of log concentration (plasma/mAb) versus percent neutralization in GraphPad Prism version 8 for macOS. For kif-grown viruses, 25 mM kifunensine was added to 293T cells on the day of transfection. To produce mutant viruses, the indicated Env-encoding plasmid (BG505, MG505.A3, or MG505.H3) was altered by site-directed mutagenesis using the QuikChange Site-Directed Mutagenesis Kit (Agilent) according to the manufacturer’s instructions. Sanger sequencing was performed to verify that each plasmid encoded the desired mutation. Mutant pseudoviruses were then produced by cotransfection with pSG3DENV as described above.

### Enzyme-linked immunosorbent assay

ELISAs were performed as previously described (*40*, *41*). Binding of rabbit mAbs was assayed by ELISA using streptavidin-coated plates to capture Avi-tagged biotinylated antigen, namely, BG505 or B41 SOSIP.664. Ninety-six–well plates were coated overnight at 4°C with streptavidin (Jackson ImmunoResearch) at 2 μg/ml in PBS. Plates were washed four times with PBS, 0.05% (v/v) Tween, and blocked with 3% (w/v) bovine serum albumin (BSA)–PBS for 1 hour. Subsequently, purified antigen (1 μg/ml) (specifically biotinylated via a C-terminal Avi-tag) was added for 2 hours. Plates were washed four times and incubated with serial dilutions of rabbit mAbs for 1 hour (either crude preparations or purified as indicated in the figure legends). Plates were then washed again, and binding was detected with anti-rabbit Fc conjugated to alkaline phosphatase (Jackson ImmunoResearch) at 1:1000 for 1 hour. For gp120 protein and the V3-FC construct, proteins were directly coated onto the ELISA plates and the 2-hour antigen capture step was omitted.

### Competition ELISA

Ninety-six–well ELISA plates were coated overnight at 4°C with mouse anti–Avi-tag antibody (GenScript) at 2 μg/ml in PBS. Plates were washed four times with PBS, 0.05% (v/v) Tween, and blocked with 3% (w/v) BSA PBS for 1 hour. Concurrently, fivefold serial dilutions of nonbiotinylated rabbit or human mAbs starting at 50 μg/ml were preincubated with purified Avi-tagged BG505 SOSIP.664 protein (1 μg/ml) for 1 hour. The mAb-SOSIP mixture was then transferred to the blocked ELISA plates and incubated for 1 hour. Plates were washed four times and incubated with biotinylated mAb (0.5 μg/ml) for 1 hour, and then washed again, and binding was detected with streptavidin–alkaline phosphatase (Jackson ImmunoResearch) at 1:1000 for 1 hour. mAbs were biotinylated using an NHS-microbiotinylation kit (Pierce). Competition is expressed as percentage binding, where 100% was the absorbance measured when BG505 SOSIP.664 protein only was captured on the anti–Avi-tag ELISA plate.

### Negative-stain EM sample preparation

43A, 43A1, and 43A2 Fabs and BG505 SOSIP.664 (±D368R/N276A) trimers were expressed in 293F cells and purified using a previously described procedure (*42*). Briefly, the trimers were affinity-purified using 2G12 antibody resin and buffer-exchanged into 20 mM tris, 0.5 M NaCl (pH 8.0; tris-buffered saline), followed by removal of aggregates, monomers, and dimers via size-exclusion chromatography. 43A2 IgG was purified by MabSelect Protein A and then digested into Fab using papain resin, followed by further purification with protein A to remove the Fc domains. Trimer-Fab complexes were formed using 6 Molar excess of Fab to trimer.

### Negative-stain EM

At a concentration of ~0.03 mg/ml, the Fab-trimer mix was applied to glow-discharged, carbon-coated 400-mesh copper grids, followed by blotting to remove excess sample. Three microliters of 2% (w/v) uranyl formate stain was applied and blotted off immediately, followed by application of another 3 μl of stain for 45 to 60 s, again followed by blotting to remove excess stain. Stained grids were allowed to air-dry and stored under ambient conditions until ready for imaging. Images were collected via Leginon software (*43*, *44*) using the Tecnai T12 electron microscope operated at 120 kV × 52,000 magnification. The electron dose was 25 e^−^/A^2^. Particles were picked from the raw images using DoG Picker (*45*) and placed into stacks using Appion software (*46*). Initial 2D reference-free alignment was performed using iterative multivariate statistical analysis/multi-reference alignments to generate a relatively clean stack of particles (*47*). Next, the clean particle stacks were converted from IMAGIC to RELION-formatted MRC stacks and subjected to RELION 2D classification (*48*), followed by RELION 3D reconstruction.

### CryoEM sample preparation

Trimer (500 μg) was incubated with 10-fold molar excess of 43A2 Fab overnight. The complex was then purified over Superose 6 column (GE Healthcare), concentrated to 1.5 mg/ml, and mixed with Lauryl Maltose Neopentyl Glycol (Anatrace) before deposition onto 2/2 Quantifoil grids (EMS) that were glow-discharged for 10 s, directly preceding the deposition in a Vitrobot (Thermo Fisher Scientific). Once sample was deposited, the grids were blotted and plunged into liquid ethane using the Vitrobot to immobilize the particles in vitreous ice. Using Leginon image acquisition software, we collected 1366 micrographs at a nominal magnification of ×29,000 with a Gatan K2 summit detector mounted on a Titan Krios set to 300 kV set to counting mode for the data collection (*44*). The dose rate was ~4.78 e^−^/pix per second with frame exposure of 250 ms, with a total exposure time and dose of 14 s and 60 e^−^/Å^2^, respectively. MotionCor2 was used for frame alignment, and CTF models were determined using GCTF (*49*). DoG Picker was used to pick 455,207 particles, which were then extracted and subsequently 2D-classified in cryoSPARC (*45*, *50*). Selected 2D classes amounting to 85,841 particles were then fed into the 3D homogeneous refinement algorithm using C3 symmetry, resulting in final resolution of ~3.52 Å.

### Model building and refinement

The BG505 SOSIP.v5.2 trimer structure from PDB (Protein Data Bank) 5ACO was docked into the cryoEM map using UCSF (University of California, San Francisco) Chimera (*51*). Subsequent iterations of manual and Rosetta fragment library–based centroid rebuilding and refinement were then performed (*52*). The resulting model was then refined using all-atom refinement under constraints of the density map. Glycans were manually built in Coot (*53*). 43A2 model was generated using Chimera-Modeller, fit into the cryoEM density, and subsequently manually built in Coot, followed by a final Rosetta refinement (*53*, *54*).

## Supplementary Material

aba0512_SM.pdf

## SUPPLEMENTARY MATERIALS

Supplementary material for this article is available at http://advances.sciencemag.org/cgi/content/full/6/23/eaba0512/DC1

## Appendix

View/request a protocol for this paper from *Bio-protocol*.

## References

[1] Hessell A. J., Malherbe D. C., Haigwood N. L., Passive and active antibody studies in primates to inform HIV vaccines. Expert Rev. Vaccines 17, 127–144 (2018). 29307225 10.1080/14760584.2018.1425619 PMC6587971

[2] Hessell A. J., Rakasz E. G., Poignard P., Hangartner L., Landucci G., Forthal D. N., Koff W. C., Watkins D. I., Burton D. R., Broadly neutralizing human anti-HIV antibody 2G12 is effective in protection against mucosal SHIV challenge even at low serum neutralizing titers. PLOS Pathog. 5, e1000433 (2009). 19436712 10.1371/journal.ppat.1000433 PMC2674935

[3] Mascola J. R., Stiegler G., VanCott T. C., Katinger H., Carpenter C. B., Hanson C. E., Beary H., Hayes D., Frankel S. S., Birx D. L., Lewis M. G., Protection of macaques against vaginal transmission of a pathogenic HIV-1/SIV chimeric virus by passive infusion of neutralizing antibodies. Nat. Med. 6, 207–210 (2000). 10655111 10.1038/72318

[4] Moldt B., Rakasz E. G., Schultz N., Chan-Hui P.-Y., Swiderek K., Weisgrau K. L., Piaskowski S. M., Bergman Z., Watkins D. I., Poignard P., Burton D. R., Highly potent HIV-specific antibody neutralization in vitro translates into effective protection against mucosal SHIV challenge in vivo. Proc. Natl. Acad. Sci. U.S.A. 109, 18921–18925 (2012). 23100539 10.1073/pnas.1214785109 PMC3503218

[5] Parren P. W., Marx P. A., Hessell A. J., Luckay A., Harouse J., Cheng-Mayer C., Moore J. P., Burton D. R., Antibody protects macaques against vaginal challenge with a pathogenic R5 simian/human immunodeficiency virus at serum levels giving complete neutralization in vitro. J. Virol. 75, 8340–8347 (2001). 11483779 10.1128/JVI.75.17.8340-8347.2001 PMC115078

[6] Pegu A., Borate B., Huang Y., Pauthner M. G., Hessell A. J., Julg B., Doria-Rose N. A., Schmidt S. D., Carpp L. N., Cully M. D., Chen X., Shaw G. M., Barouch D. H., Haigwood N. L., Corey L., Burton D. R., Roederer M., Gilbert P. B., Mascola J. R., Huang Y., A meta-analysis of passive immunization studies shows that serum-neutralizing antibody titer associates with protection against SHIV challenge. Cell Host Microbe 26, 336–346.e3 (2019). 31513771 10.1016/j.chom.2019.08.014 PMC6755677

[7] Burton D. R., Advancing an HIV vaccine; advancing vaccinology. Nat. Rev. Immunol. 19, 77–78 (2019). 30560910 10.1038/s41577-018-0103-6 PMC6425752

[8] Sanders R. W., Moore J. P., Native-like Env trimers as a platform for HIV-1 vaccine design. Immunol. Rev. 275, 161–182 (2017). 28133806 10.1111/imr.12481 PMC5299501

[9] Andrabi R., Bhiman J. N., Burton D. R., Strategies for a multi-stage neutralizing antibody-based HIV vaccine. Curr. Opin. Immunol. 53, 143–151 (2018). 29775847 10.1016/j.coi.2018.04.025 PMC6141341

[10] Sanders R. W., Derking R., Cupo A., Julien J.-P., Yasmeen A., de Val N., Kim H. J., Blattner C., de la Peña A. T., Korzun J., Golabek M., de los Reyes K., Ketas T. J., van Gils M. J., King C. R., Wilson I. A., Ward A. B., Klasse P. J., Moore J. P., A next-generation cleaved, soluble HIV-1 env trimer, BG505 SOSIP.664 gp140, expresses multiple epitopes for broadly neutralizing but not non-neutralizing antibodies. PLOS Pathog. 9, e1003618 (2013). 24068931 10.1371/journal.ppat.1003618 PMC3777863

[11] Cheng C., Pancera M., Bossert A., Schmidt S. D., Chen R. E., Chen X., Druz A., Narpala S., Doria-Rose N. A., McDermott A. B., Kwong P. D., Mascola J. R., Immunogenicity of a prefusion HIV-1 envelope trimer in complex with a quaternary-structure-specific antibody. J. Virol. 90, 2740–2755 (2015). 26719262 10.1128/JVI.02380-15 PMC4810637

[12] De Taeye S. W., Ozorowski G., De La Peña A. T., Guttman M., Julien J.-P., Van Den Kerkhof T. L. G. M., Burger J. A., Pritchard L. K., Pugach P., Yasmeen A., Crampton J., Hu J., Bontjer I., Torres J. L., Arendt H., Destefano J., Koff W. C., Schuitemaker H., Eggink D., Berkhout B., Dean H., Labranche C., Crotty S., Crispin M., Montefiori D. C., Klasse P. J., Lee K. K., Moore J. P., Wilson I. A., Ward A. B., Sanders R. W., Immunogenicity of stabilized HIV-1 envelope trimers with reduced exposure of non-neutralizing epitopes. Cell 163, 1702–1715 (2015). 26687358 10.1016/j.cell.2015.11.056 PMC4732737

[13] Feng Y., Tran K., Bale S., Kumar S., Guenaga J., Wilson R., de Val N., Arendt H., DeStefano J., Ward A. B., Wyatt R. T., Thermostability of well-ordered HIV spikes correlates with the elicitation of autologous tier 2 neutralizing antibodies. PLOS Pathog. 12, e1005767 (2016). 27487086 10.1371/journal.ppat.1005767 PMC4972253

[14] Klasse P. J., LaBranche C. C., Ketas T. J., Ozorowski G., Cupo A., Pugach P., Ringe R. P., Golabek M., van Gils M. J., Guttman M., Lee K. K., Wilson I. A., Butera S. T., Ward A. B., Montefiori D. C., Sanders R. W., Moore J. P., Sequential and simultaneous immunization of rabbits with HIV-1 envelope glycoprotein SOSIP.664 trimers from clades A, B and C. PLOS Pathog. 12, e1005864 (2016). 27627672 10.1371/journal.ppat.1005864 PMC5023125

[15] Martinez-Murillo P., Tran K., Guenaga J., Lindgren G., Àdori M., Feng Y., Phad G. E., Vázquez Bernat N., Bale S., Ingale J., Dubrovskaya V., O’Dell S., Pramanik L., Spångberg M., Corcoran M., Loré K., Mascola J. R., Wyatt R. T., Karlsson Hedestam G. B., Particulate array of well-ordered HIV clade C Env trimers elicits neutralizing antibodies that display a unique V2 cap approach. Immunity 46, 804–817.e7 (2017). 28514687 10.1016/j.immuni.2017.04.021 PMC5528178

[16] Pauthner M., Havenar-Daughton C., Sok D., Nkolola J. P., Bastidas R., Boopathy A. V., Carnathan D. G., Chandrashekar A., Cirelli K. M., Cottrell C. A., Eroshkin A. M., Guenaga J., Kaushik K., Kulp D. W., Liu J., McCoy L. E., Oom A. L., Ozorowski G., Post K. W., Sharma S. K., Steichen J. M., de Taeye S. W., Tokatlian T., de la Peña A. T., Butera S. T., LaBranche C. C., Montefiori D. C., Silvestri G., Wilson I. A., Irvine D. J., Sanders R. W., Schief W. R., Ward A. B., Wyatt R. T., Barouch D. H., Crotty S., Burton D. R., Elicitation of robust tier 2 neutralizing antibody responses in nonhuman primates by HIV envelope trimer immunization using optimized approaches. Immunity 46, 1073–1088.e6 (2017). 28636956 10.1016/j.immuni.2017.05.007 PMC5483234

[17] Saunders K. O., Verkoczy L. K., Jiang C., Zhang J., Parks R., Chen H., Housman M., Bouton-Verville H., Shen X., Trama A. M., Scearce R., Sutherland L., Santra S., Newman A., Eaton A., Xu K., Georgiev I. S., Joyce M. G., Tomaras G. D., Bonsignori M., Reed S. G., Salazar A., Mascola J. R., Moody M. A., Cain D. W., Centlivre M., Zurawski S., Zurawski G., Erickson H. P., Kwong P. D., Alam S. M., Levy Y., Montefiori D. C., Haynes B. F., Vaccine induction of heterologous tier 2 HIV-1 neutralizing antibodies in animal models. Cell Rep. 21, 3681–3690 (2017). 29281818 10.1016/j.celrep.2017.12.028 PMC5777169

[18] McCoy L. E., van Gils M. J., Ozorowski G., Messmer T., Briney B., Voss J. E., Kulp D. W., Macauley M. S., Sok D., Pauthner M., Menis S., Cottrell C. A., Torres J. L., Hsueh J., Schief W. R., Wilson I. A., Ward A. B., Sanders R. W., Burton D. R., Holes in the glycan shield of the native HIV envelope are a target of trimer-elicited neutralizing antibodies. Cell Rep. 16, 2327–2338 (2016). 27545891 10.1016/j.celrep.2016.07.074 PMC5007210

[19] Bianchi M., Turner H. L., Nogal B., Cottrell C. A., Oyen D., Pauthner M., Bastidas R., Nedellec R., McCoy L. E., Wilson I. A., Burton D. R., Ward A. B., Hangartner L., Electron-microscopy-based epitope mapping defines specificities of polyclonal antibodies elicited during HIV-1 BG505 envelope trimer immunization. Immunity 49, 288–300.e8 (2018). 30097292 10.1016/j.immuni.2018.07.009 PMC6104742

[20] Wagh K., Kreider E. F., Li Y., Barbian H. J., Learn G. H., Giorgi E., Hraber P. T., Decker T. G., Smith A. G., Gondim M. V., Gillis L., Wandzilak J., Chuang G.-Y., Rawi R., Cai F., Pellegrino P., Williams I., Overbaugh J., Gao F., Kwong P. D., Haynes B. F., Shaw G. M., Borrow P., Seaman M. S., Hahn B. H., Korber B., Completeness of HIV-1 envelope glycan shield at transmission determines neutralization breadth. Cell Rep. 25, 893–908.e7 (2018). 30355496 10.1016/j.celrep.2018.09.087 PMC6426304

[21] Julien J.-P., Sok D., Khayat R., Lee J. H., Doores K. J., Walker L. M., Ramos A., Diwanji D. C., Pejchal R., Cupo A., Katpally U., Depetris R. S., Stanfield R. L., McBride R., Marozsan A. J., Paulson J. C., Sanders R. W., Moore J. P., Burton D. R., Poignard P., Ward A. B., Wilson I. A., Broadly neutralizing antibody PGT121 allosterically modulates CD4 binding via recognition of the HIV-1 gp120 V3 base and multiple surrounding glycans. PLOS Pathog. 9, e1003342 (2013). 23658524 10.1371/journal.ppat.1003342 PMC3642082

[22] Sok D., Pauthner M., Briney B., Lee J. H., Saye-Francisco K. L., Hsueh J., Ramos A., Le K. M., Jones M., Jardine J. G., Bastidas R., Sarkar A., Liang C.-H., Shivatare S. S., Wu C.-Y., Schief W. R., Wong C.-H., Wilson I. A., Ward A. B., Zhu J., Poignard P., Burton D. R., A prominent site of antibody vulnerability on HIV envelope incorporates a motif associated with CCR5 binding and its camouflaging glycans. Immunity 45, 31–45 (2016). 27438765 10.1016/j.immuni.2016.06.026 PMC4990068

[23] Sok D., Doores K. J., Briney B., Le K. M., Saye-Francisco K. L., Ramos A., Kulp D. W., Julien J.-P., Menis S., Wickramasinghe L., Seaman M. S., Schief W. R., Wilson I. A., Poignard P., Burton D. R., Promiscuous glycan site recognition by antibodies to the high-mannose patch of gp120 broadens neutralization of HIV. Sci. Transl. Med. 6, 236ra63 (2014). 10.1126/scitranslmed.3008104 PMC4095976 24828077

[24] Deshpande S., Patil S., Kumar R., Hermanus T., Murugavel K. G., Srikrishnan A. K., Solomon S., Morris L., Bhattacharya J., HIV-1 clade C escapes broadly neutralizing autologous antibodies with N332 glycan specificity by distinct mechanisms. Retrovirology 13, 60 (2016). 27576440 10.1186/s12977-016-0297-2 PMC5006590

[25] Garces F., Sok D., Kong L., McBride R., Kim H. J., Saye-Francisco K. F., Julien J.-P., Hua Y., Cupo A., Moore J. P., Paulson J. C., Ward A. B., Burton D. R., Wilson I. A., Structural evolution of glycan recognition by a family of potent HIV antibodies. Cell 159, 69–79 (2014). 25259921 10.1016/j.cell.2014.09.009 PMC4278586

[26] Kong L., Lee J. H., Doores K. J., Murin C. D., Julien J.-P., McBride R., Liu Y., Marozsan A., Cupo A., Klasse P.-J., Hoffenberg S., Caulfield M., King C. R., Hua Y., Le K. M., Khayat R., Deller M. C., Clayton T., Tien H., Feizi T., Sanders R. W., Paulson J. C., Moore J. P., Stanfield R. L., Burton D. R., Ward A. B., Wilson I. A., Supersite of immune vulnerability on the glycosylated face of HIV-1 envelope glycoprotein gp120. Nat. Struct. Mol. Biol. 20, 796–803 (2013). 23708606 10.1038/nsmb.2594 PMC3823233

[27] Barnes C. O., Gristick H. B., Freund N. T., Escolano A., Lyubimov A. Y., Hartweger H., West Jr A. P., Cohen A. E., Nussenzweig M. C., Bjorkman P. J., Structural characterization of a highly-potent V3-glycan broadly neutralizing antibody bound to natively-glycosylated HIV-1 envelope. Nat. Commun. 9, 1251 (2018). 29593217 10.1038/s41467-018-03632-y PMC5871869

[28] Wu X., Parast A. B., Richardson B. A., Nduati R., John-Stewart G., Mbori-Ngacha D., Rainwater S. M. J., Overbaugh J., Neutralization escape variants of human immunodeficiency virus type 1 are transmitted from mother to infant. J. Virol. 80, 835–844 (2006). 16378985 10.1128/JVI.80.2.835-844.2006 PMC1346878

[29] Lyumkis D., Julien J.-P., de Val N., Cupo A., Potter C. S., Klasse P.-J., Burton D. R., Sanders R. W., Moore J. P., Carragher B., Wilson I. A., Ward A. B., Cryo-EM structure of a fully glycosylated soluble cleaved HIV-1 envelope trimer. Science 342, 1484–1490 (2013). 24179160 10.1126/science.1245627 PMC3954647

[30] Lee J. H., de Val N., Lyumkis D., Ward A. B., Model building and refinement of a natively glycosylated HIV-1 Env protein by high-resolution cryoelectron microscopy. Structure 23, 1943–1951 (2015). 26388028 10.1016/j.str.2015.07.020 PMC4618500

[31] Lee J. H., Andrabi R., Su C.-Y., Yasmeen A., Julien J.-P., Kong L., Wu N. C., McBride R., Sok D., Pauthner M., Cottrell C. A., Nieusma T., Blattner C., Paulson J. C., Klasse P. J., Wilson I. A., Burton D. R., Ward A. B., A broadly neutralizing antibody targets the dynamic HIV envelope trimer apex via a long, rigidified, and anionic β-hairpin structure. Immunity 46, 690–702 (2017). 28423342 10.1016/j.immuni.2017.03.017 PMC5400778

[32] Nogal B., Bianchi M., Cottrell C. A., Kirchdoerfer R. N., Sewall M., Turner H. L., Zhao F., Sok D., Burton D. R., Hangartner L., Ward A. B., Mapping polyclonal antibody responses in non-human primates vaccinated with HIV Env trimer subunit vaccines. Cell Rep. **30**, 3755–3765.e7 (2020). 10.1016/j.celrep.2020.02.061 PMC7153566 32187547

[33] Julien J.-P., Cupo A., Sok D., Stanfield R. L., Lyumkis D., Deller M. C., Klasse P.-J., Burton D. R., Sanders R. W., Moore J. P., Ward A. B., Wilson I. A., Crystal structure of a soluble cleaved HIV-1 envelope trimer. Science 342, 1477–1483 (2013). 24179159 10.1126/science.1245625 PMC3886632

[34] Steichen J. M., Kulp D. W., Tokatlian T., Escolano A., Dosenovic P., Stanfield R. L., McCoy L. E., Ozorowski G., Hu X., Kalyuzhniy O., Briney B., Schiffner T., Garces F., Freund N. T., Gitlin A. D., Menis S., Georgeson E., Kubitz M., Adachi Y., Jones M., Mutafyan A. A., Yun D. S., Mayer C. T., Ward A. B., Burton D. R., Wilson I. A., Irvine D. J., Nussenzweig M. C., Schief W. R., HIV vaccine design to target germline precursors of glycan-dependent broadly neutralizing antibodies. Immunity 45, 483–496 (2016). 27617678 10.1016/j.immuni.2016.08.016 PMC5040827

[35] Escolano A., Gristick H. B., Abernathy M. E., Merkenschlager J., Gautam R., Oliveira T. Y., Pai J., West Jr. A. P., Barnes C. O., Cohen A. A., Wang H., Golijanin J., Yost D., Keeffe J. R., Wang Z., Zhao P., Yao K.-H., Bauer J., Nogueira L., Gao H., Voll A. V., Montefiori D. C., Seaman M. S., Gazumyan A., Silva M., McGuire A. T., Stamatatos L., Irvine D. J., Wells L., Martin M. A., Bjorkman P. J., Nussenzweig M. C., Immunization expands B cells specific to HIV-1 V3 glycan in mice and macaques. Nature 570, 468–473 (2019). 31142836 10.1038/s41586-019-1250-z PMC6657810

[36] Klasse P. J., Ketas T. J., Cottrell C. A., Ozorowski G., Debnath G., Camara D., Francomano E., Pugach P., Ringe R. P., LaBranche C. C., van Gils M. J., Bricault C. A., Barouch D. H., Crotty S., Silvestri G., Kasturi S., Pulendran B., Wilson I. A., Montefiori D. C., Sanders R. W., Ward A. B., Moore J. P., Epitopes for neutralizing antibodies induced by HIV-1 envelope glycoprotein BG505 SOSIP trimers in rabbits and macaques. PLOS Pathog. 14, e1006913 (2018). 29474444 10.1371/journal.ppat.1006913 PMC5841823

[37] Escolano A., Steichen J. M., Dosenovic P., Kulp D. W., Golijanin J., Sok D., Freund N. T., Gitlin A. D., Oliveira T., Araki T., Lowe S., Chen S. T., Heinemann J., Yao K.-H., Georgeson E., Saye-Francisco K. L., Gazumyan A., Adachi Y., Kubitz M., Burton D. R., Schief W. R., Nussenzweig M. C., Sequential immunization elicits broadly neutralizing anti-HIV-1 antibodies in Ig knockin mice. Cell 166, 1445–1458.e12 (2016). 27610569 10.1016/j.cell.2016.07.030 PMC5019122

[38] Li M., Gao F., Mascola J. R., Stamatatos L., Polonis V. R., Koutsoukos M., Voss G., Goepfert P., Gilbert P., Greene K. M., Bilska M., Kothe D. L., Salazar-Gonzalez J. F., Wei X., Decker J. M., Hahn B. H., Montefiori D. C., Human immunodeficiency virus type 1 *env* clones from acute and early subtype B infections for standardized assessments of vaccine-elicited neutralizing antibodies. J. Virol. 79, 10108–10125 (2005). 16051804 10.1128/JVI.79.16.10108-10125.2005 PMC1182643

[39] Seaman M. S., Janes H., Hawkins N., Grandpre L. E., Devoy C., Giri A., Coffey R. T., Harris L., Wood B., Daniels M. G., Bhattacharya T., Lapedes A., Polonis V. R., McCutchan F. E., Gilbert P. B., Self S. G., Korber B. T., Montefiori D. C., Mascola J. R., Tiered categorization of a diverse panel of HIV-1 Env pseudoviruses for assessment of neutralizing antibodies. J. Virol. 84, 1439–1452 (2010). 19939925 10.1128/JVI.02108-09 PMC2812321

[40] Sanders R. W., van Gils M. J., Derking R., Sok D., Ketas T. J., Burger J. A., Ozorowski G., Cupo A., Simonich C., Goo L., Arendt H., Kim H. J., Lee J. H., Pugach P., Williams M., Debnath G., Moldt B., van Breemen M. J., Isik G., Medina-Ramírez M., Back J. W., Koff W. C., Julien J.-P., Rakasz E. G., Seaman M. S., Guttman M., Lee K. K., Klasse P. J., LaBranche C., Schief W. R., Wilson I. A., Overbaugh J., Burton D. R., Ward A. B., Montefiori D. C., Dean H., Moore J. P., HIV-1 VACCINES. HIV-1 neutralizing antibodies induced by native-like envelope trimers. Science 349, aac4223 (2015). 26089353 10.1126/science.aac4223 PMC4498988

[41] Derking R., Ozorowski G., Sliepen K., Yasmeen A., Cupo A., Torres J. L., Julien J.-P., Lee J. H., van Montfort T., de Taeye S. W., Connors M., Burton D. R., Wilson I. A., Klasse P.-J., Ward A. B., Moore J. P., Sanders R. W., Comprehensive antigenic map of a cleaved soluble HIV-1 envelope trimer. PLOS Pathog. 11, e1004767 (2015). 25807248 10.1371/journal.ppat.1004767 PMC4373910

[42] Lee J. H., Leaman D. P., Kim A. S., Torrents de la Peña A., Sliepen K., Yasmeen A., Derking R., Ramos A., de Taeye S. W., Ozorowski G., Klein F., Burton D. R., Nussenzweig M. C., Poignard P., Moore J. P., Klasse P. J., Sanders R. W., Zwick M. B., Wilson I. A., Ward A. B., Antibodies to a conformational epitope on gp41 neutralize HIV-1 by destabilizing the Env spike. Nat. Commun. 6, 8167 (2015). 26404402 10.1038/ncomms9167 PMC4586043

[43] Carragher B., Kisseberth N., Kriegman D., Milligan R. A., Potter C. S., Pulokas J., Reilein A., Leginon: An automated system for acquisition of images from vitreous ice specimens. J. Struct. Biol. 132, 33–45 (2000). 11121305 10.1006/jsbi.2000.4314

[44] Suloway C., Pulokas J., Fellmann D., Cheng A., Guerra F., Quispe J., Stagg S., Potter C. S., Carragher B., Automated molecular microscopy: The new Leginon system. J. Struct. Biol. 151, 41–60 (2005). 15890530 10.1016/j.jsb.2005.03.010

[45] Voss N. R., Yoshioka C. K., Radermacher M., Potter C. S., Carragher B., DoG Picker and TiltPicker: Software tools to facilitate particle selection in single particle electron microscopy. J. Struct. Biol. 166, 205–213 (2009). 19374019 10.1016/j.jsb.2009.01.004 PMC2768396

[46] Lander G. C., Stagg S. M., Voss N. R., Cheng A., Fellmann D., Pulokas J., Yoshioka C., Irving C., Mulder A., Lau P.-W., Lyumkis D., Potter C. S., Carragher B., Appion: An integrated, database-driven pipeline to facilitate EM image processing. J. Struct. Biol. 166, 95–102 (2009). 19263523 10.1016/j.jsb.2009.01.002 PMC2775544

[47] Ogura T., Iwasaki K., Sato C., Topology representing network enables highly accurate classification of protein images taken by cryo electron-microscope without masking. J. Struct. Biol. 143, 185–200 (2003). 14572474 10.1016/j.jsb.2003.08.005

[48] Scheres S. H. W., RELION: Implementation of a Bayesian approach to cryo-EM structure determination. J. Struct. Biol. 180, 519–530 (2012). 23000701 10.1016/j.jsb.2012.09.006 PMC3690530

[49] Zheng S. Q., Palovcak E., Armache J.-P., Verba K. A., Cheng Y., Agard D. A., MotionCor2: Anisotropic correction of beam-induced motion for improved cryo-electron microscopy. Nat. Methods 14, 331–332 (2017). 28250466 10.1038/nmeth.4193 PMC5494038

[50] Punjani A., Rubinstein J. L., Fleet D. J., Brubaker M. A., cryoSPARC: Algorithms for rapid unsupervised cryo-EM structure determination. Nat. Methods 14, 290–296 (2017). 28165473 10.1038/nmeth.4169

[51] Pettersen E. F., Goddard T. D., Huang C. C., Couch G. S., Greenblatt D. M., Meng E. C., Ferrin T. E., UCSF Chimera A visualization system for exploratory research and analysis. J. Comput. Chem. 25, 1605–1612 (2004). 15264254 10.1002/jcc.20084

[52] Simoncini D., Berenger F., Shrestha R., Zhang K. Y. J., A Probabilistic fragment-based protein structure prediction algorithm. PLOS One. 7, e38799 (2012). 22829868 10.1371/journal.pone.0038799 PMC3400640

[53] Emsley P., Lohkamp B., Scott W. G., Cowtan K., Features and development of *Coot*. Acta Crystallogr. D Biol. Crystallogr. 66, 486–501 (2010). 20383002 10.1107/S0907444910007493 PMC2852313

[54] Yang Z., Lasker K., Schneidman-Duhovny D., Webb B., Huang C. C., Pettersen E. F., Goddard T. D., Meng E. C., Sali A., Ferrin T. E., UCSF Chimera, MODELLER, and IMP: An integrated modeling system. J. Struct. Biol. 179, 269–278 (2012). 21963794 10.1016/j.jsb.2011.09.006 PMC3410985

