# Supplementary material for: HIV envelope trimer-elicited autologous neutralizing antibodies bind a region overlapping the N332 glycan supersite
Source: Sci Adv. 2020 Jun 5;6(23):eaba0512. doi: 10.1126/sciadv.aba0512 (PMC7274786; doi:10.1126/sciadv.aba0512)
Supplement: aba0512_SM.pdf [file aba0512_SM.pdf]

Science Advances
AAAS

advances.sciencemag.org/cgi/content/full/6/23/eaba0512/DC1

Supplementary Materials for

HIV envelope trimer-elicited autologous neutralizing antibodies bind a region overlapping the N332 glycan supersite

Bartek Nogal, Laura E. McCoy, Marit J. van Gils, Christopher A. Cottrell, James E. Voss, Raiees Andrabi, Matthias Pauthner, Chi-Hui Liang, Terrence Messmer, Rebecca Nedellec, Mia Shin, Hannah L. Turner, Gabriel Ozorowski, Rogier W. Sanders, Dennis R. Burton*, Andrew B. Ward*

*Corresponding author. Email: burton@scripps.edu (D.R.B.); andrew@scripps.edu (A.B.W.)

Published 5 June 2020, *Sci. Adv.* **6**, eaba0512 (2020)
DOI: 10.1126/sciadv.aba0512

This PDF file includes:

Figs. S1 to S5
Tables S1 and S2

Figure S1

```
43A2HC   SQLVESGGGLVQPEGSLTLTCKASGFSFSRSQYMCWVRQAPGKGLEWITCVYPDDDTPYY
43A1HC   SQLVESGGGLVKPGASLTLTCKASGFSFSRQNYMCWVRQAPGKGLEWVACIYTDDGTRYY
43AHC    SQLVESGGGLVKPGTSLTLTCKASGFSFSRQNYMCWVRQAPGKGLEWVACIYTDDGTRYY
         ***********:*  ***************.:***************::*:* ** * **

43A2HC   ATWAKGRFTISKTSSTTVTLRLTSLTEADTATYFCARTSGFGGYSYAAHGVDLWGPGTLV
43A1HC   ANWAKGRFTLSNPSSTTVTLQMTGLKAADTATYFCARTSGYGGYSYAAYGIDLWGPGTLV
43AHC    ASWAKGRLTLSNPSSTTVTLQMTSLTAADTATYFCARTSGYGGYSYAAYGIDLWGPGTLV
         *.*****:*:*: *******::*.*. *************:*******:*:*********

43A2HC   TVSS
43A1HC   TVSS
43AHC    TVSS
         ****

43A2KC   DIVMTQTPASVSEPVGGTVTIKCQASHNIRSYLSWYQQKVGQPPKRLIYETSNLASGVPS
43A1KC   DIVMTQTPASVSEPVGGTVTIKCQASQSISSYLSWYQQKPGQPPKRLIYGASTLASGVPS
43AKC    DIVMTQTPASVSEPVGGTVTIKCQASQSISSYLSWYQQKPGQPPKRLIYGAAALASGVPS
         **************************:.* ********* ********* :: *******

43A2KC   RFAGSGSGTEFTLTISDLECADAATYYCQSNFGLSDSRTYNFGGGTEVVVK
43A1KC   RFKGSGSGTEYTLTISDLECADAATYYCQSNFGLSDSRTYPFGGGTEVVVK
43AKC    RFKGSGSGARFTLTISDLECADAATYYCQSNFGLSDSRTYPFGGGTEMVVK
         ** *****:.:***************************** ******:***
```

Fig. S1. Related to Fig. 1. Sequence alignment of the heavy and light chains of the 43A monoclonal antibodies.

Figure S2

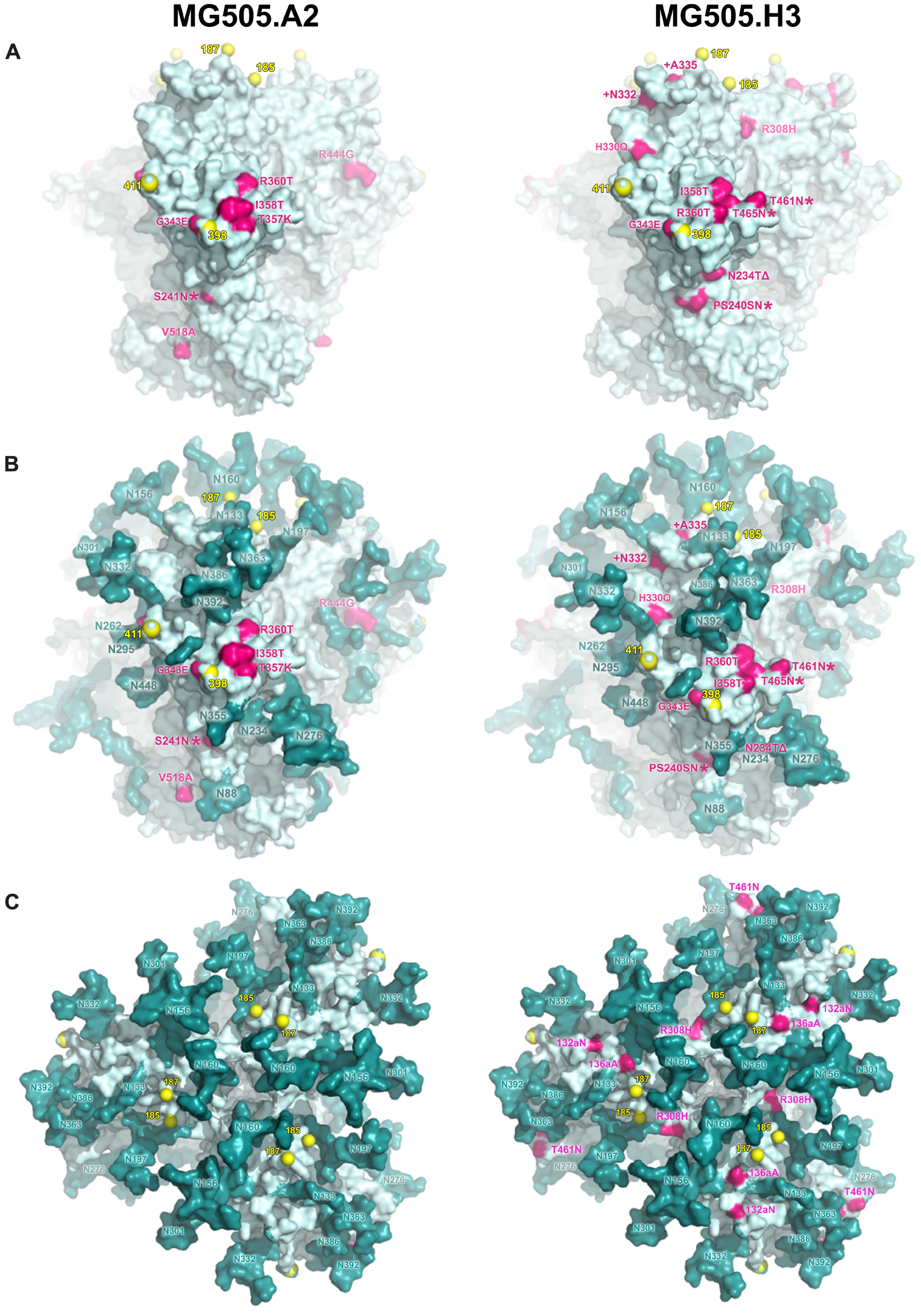

D

	130 … 140	185 … 187	398 … 411
BG505 -	Q C T - N V T N - N I T D	N E N Q G N R S N N S	N T S V Q G S N S T E S N
MG505.A2 -	Q C T - N V T N - N I T D	N E N Q G N G S N N S	N T S V Q G S N S T G S N
MG505.H3 -	Q C T N N V T N A N I T D	N E N Q G N S S K S S	N T S V Q G S N S T G S N

Fig. S2. Related to Fig. 3. Comparison of MG505.A2 and MG505.H3 Env. (A) MG505.A2 (left-hand panel) and MG505.H3 (right-hand panel) amino acid sequences modelled onto the structure of crystal structure of BG505 SOSIP.664 (PDB: 53TX). Residues colored pink differ between MG505.A2 and BG505 (left-hand panel) and between MG505.H3 and BG505 (right-hand panel). Residues colored yellow mark the limit of the predicted structures (between yellow spheres are unstructured loops). (**B**) Same models as in (**A**) but with N-linked glycans are highlighted in teal and numbered. (**C**) Apex view of the structures shown in (**B**). (**D**)An alignment of sequence changes between MG505.A2 and MG505.H3.

Figure S3

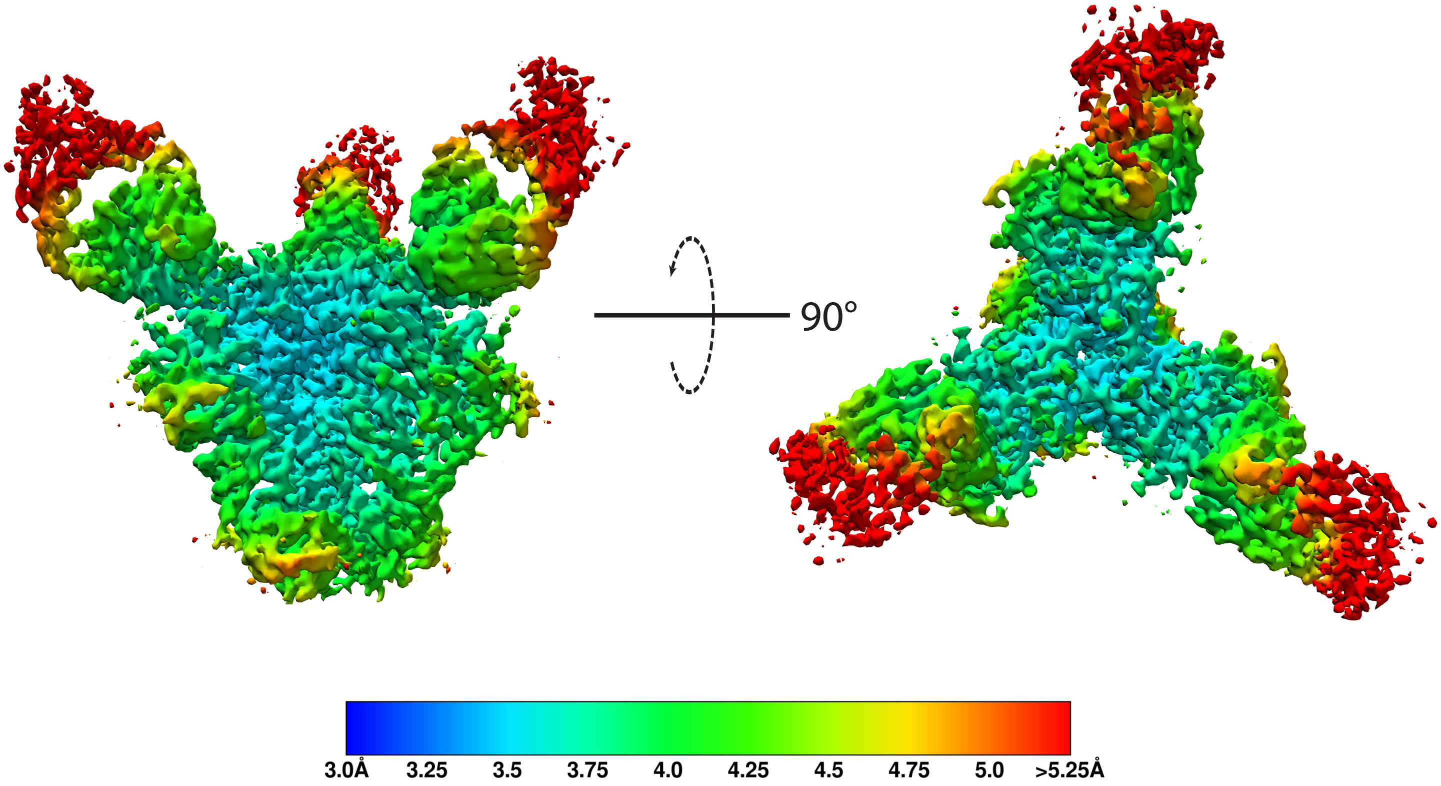

Fig. S3. Related to Fig. 5. Local resolution maps of the complex.

Figure S4

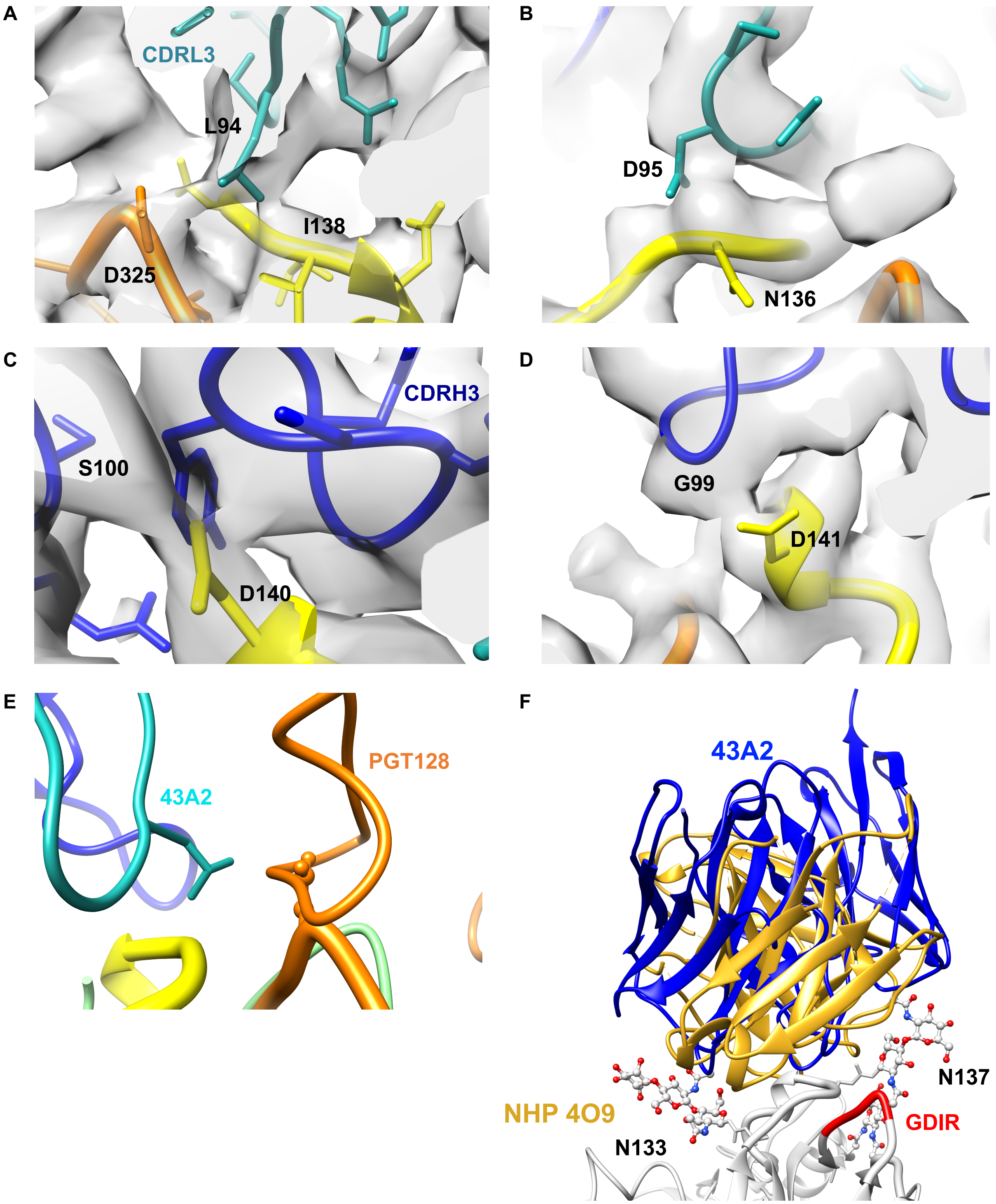

Fig. S4. Related to Fig. 5. CryoEM map details of the 43A2– BG505 SOSIP.v5.2 interactions. (**A**) and (**B**) CDRL3 interactions with the V1 and V3 loops, showing L94 making contact with the GDIR motif, also showing proximity of the V1 loop (yellow) to the GDIR motif (orange) (**C**) and (**D**) CDRH3-V1 interactions and (**E**) Close-up of competition for the V3 residue D325 (thick orange loop) between PGT128 (orange) and 43A2 (cyan) (**F**) Overlay of 43A2 and non-human primate V1 loop binding polyclonal antibodies from animal 4O9, showing how both the rabbit monoclonal antibody and the NHP polyclonal antibodies avoid glycans in order to bind the N332 glycan supersite region on gp120 (32).

Figure S5

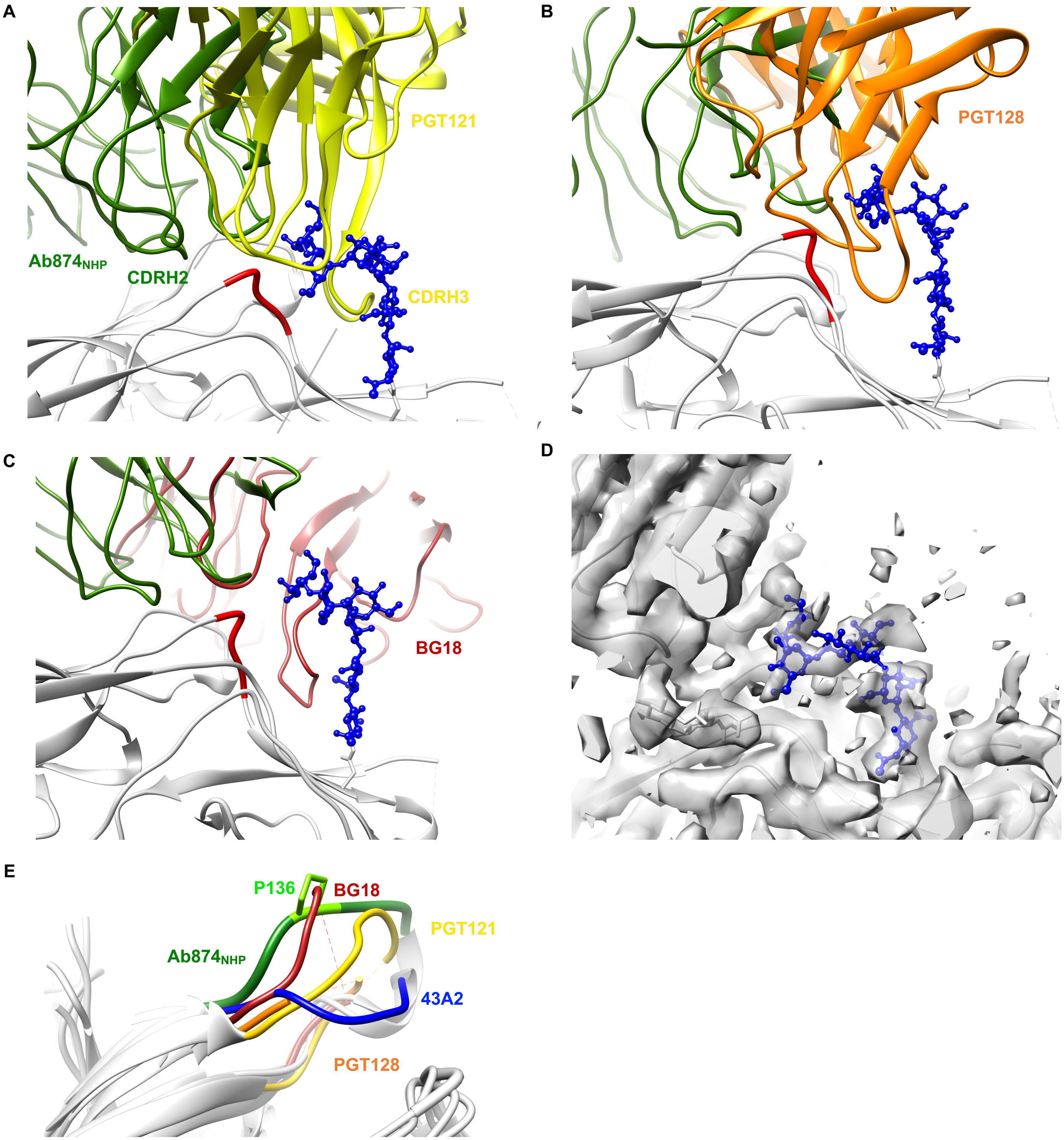

Fig. S5. Related to Fig. 6. Comparison of N332 glycan interactions between bnAbs and Ab874$_{NHP}$(35) (**A**) , (**B**) and (**C**) Ab874$_{NHP}$'s relatively short CDRH2 is positioned to potentially make minor contacts with an N332 glycan terminal mannose while bnAbs PGT121 (PDB: 5CEZ), PGT128 (PDB: 5ACO), and B18 (PDB: 6CH7), respectively, make both explicit and substantial contacts with both the N332 glycan and N332 (PGT121 and BG18) using their long CDH3's, **(D)** The N332 glycan is not well resolved in the Ab874$_{NHP}$ bound trimer structure, consistent with it not making any substantial contact with the antibody. (**E**) Comparison of V1 loop conformations in the ground state (43A2) and states induced by bnAbs or via a N136P mutation (Ab874$_{NHP}$).

Table S1. Related to Figs. 5 and 6. CryoEM model building and statistics.

EM Data Collection Parameters	
Complex	BG505 SOSIP.v5.2 + 43A2
Microscope	Titan Krios
Voltage, kV	300
Detector	Gatan K2 Sumit
Recording Mode	Counting
Magnification	29,000
Moive micrograph pixel size, Å	1.03
Dose rate, e^-/[(camera pixel)*s]	4.78
No. of frames per moive micrograph	44
Frame exposure time, ms	250
Movie micrograph exposure time, s	11
Total dose, e^-/Å^2	49.6
Defocus range, μm	0.60 to 2.00

Map and Model Refinement Parameters	
No. of movie micrographs	1366
No. of molecular projection images in map	85841
Symmetry	C3
Map resolution (FSC 0.143)	3.52
Map sharpening B-factor, Å^2	-144.1
No. of atoms in deposited model	19596
MolProbity score	1.21
Cβ Outliers (%)	0.00
Rotamer Outliers (%)	0.00
Rama Outliers (%)	0.78
Clashscore	1.60
EMRinger score	2.84
Privateer	pass
PDB-CARE	pass
CARP	pass
EMDB	6VO0
PDB ID	EMD-21256

Table S2. Related to Fig. 6. Ab874$_{NHP}$, Ab897$_{NHP}$, 43A2 BG505 epitope contacts vs N332 glycan supersite bnAbs. Antibody-gp120 side chain contacts (with radius < 4.0 Å) of mAbs derived from immunized NHPs and rabbits primarily interact with the V1/V2 regions while predominant interactions by bnAbs are with the V3 base (based on PDBs: 6ORO, 6ORP, 5CEZ, 5ACO, 6CH7 corresponding to Ab874$_{NHP}$, Ab897$_{NHP}$, PGT121, PGT128, and BG18). Red type indicates non-native residues

gp120 region	Residue number	Ab874$_{NHP}$	Ab897$_{NHP}$	PGT121	PGT128	BG18	43A
V1	132	T	T				
	133	N					
	134	Y	Y				
	135	A	A	T			T
	136	P	P	N			N
	137		N	A	N	N	N
	138					I	I
	139					T	T
	140						D
	141						D
	142						
	143						R
	156	Q	Q				
V2	171	K	K				
	173	Y	Y				
	188		N				
V3	295					N	
	301				N		
	305		K				
	321	D	D		D		
	322	I	I	I	I		
	323		I	I	I		
	324	G	G	G	G		
	325	D		D	D	D	D
	326	I		I	I	I	
	327			R	R	R	
	328			Q		Q	
	330			H		H	
	332			N		N	
V4	413			S			
	415			T			
	417			P			
	442				V		
	444			R	R		
